# Stratifying esophago-gastric cancer treatment using a patient-derived organoid-based threshold

**DOI:** 10.1186/s12943-023-01919-3

**Published:** 2024-01-10

**Authors:** Tim Schmäche, Juliane Fohgrub, Anna Klimova, Karin Laaber, Stephan Drukewitz, Felix Merboth, Alexander Hennig, Therese Seidlitz, Friederike Herbst, Franziska Baenke, Anne-Marlen Ada, Thomas Groß, Carina Wenzel, Claudia R. Ball, Christian Praetorius, Thomas Schmidt, Barbara Ringelband-Schilling, Ronald Koschny, Albrecht Stenzinger, Ingo Roeder, Dirk Jaeger, Sebastian Zeissig, Thilo Welsch, Daniela Aust, Hanno Glimm, Gunnar Folprecht, Jürgen Weitz, Georg M. Haag, Daniel E. Stange

**Affiliations:** 1 Department of Visceral, Thoracic and Vascular Surgery, Medical Faculty and University Hospital Carl Gustav Carus, Technische Universität Dresden, Fetscherstraße 74, Dresden, 01307 Germany; 2 National Center for Tumor Diseases Dresden (NCT/UCC), a partnership between DKFZ, Faculty of Medicine and University Hospital Carl Gustav Carus, TUD Dresden University of Technology, and Helmholtz-Zentrum Dresden - Rossendorf (HZDR), Dresden, Germany; 3 Institute for Medical Informatics and Biometry, Faculty of Medicine, Technische Universität Dresden, Dresden, Germany; 4 German Cancer Research Center (DKFZ) Heidelberg, Translational Functional Cancer Genomics, Heidelberg, Germany; 5 Faculty of Biosciences, Heidelberg University, Heidelberg, Germany; 6 Core Unit for Molecular Tumor Diagnostics (CMTD), Technical University Dresden, Dresden, Germany; 7 Institute of Human Genetics, University of Leipzig Medical Center, Leipzig, Germany; 8 Center for Cellular Immunotherapies, Perelman School of Medicine, University of Pennsylvania, Philadelphia, PA USA; 9 Abramson Cancer Center, Perelman School of Medicine, University of Pennsylvania, Philadelphia, PA USA; 10 German Cancer Consortium (DKTK), Dresden, Germany; 11 Institute of Pathology, University Hospital Carl Gustav CarusTechnische Universität Dresden, Dresden, Germany; 12 Department for Translational Medical Oncology, National Center for Tumor Diseases Dresden (NCT/UCC), Dresden, Germany; 13 Translational Medical Oncology, Faculty of Medicine and University Hospital Carl Gustav CarusTechnische Universität Dresden, Dresden, Germany; 14 German Cancer Consortium (DKTK), partner side Dresden, Dresden, Germany; 15 TUD Dresden University of Technology, Faculty of Biology, Technische Universität Dresden, Dresden, Germany; 16 Department of General, Visceral and Transplantation Surgery, University Hospital of Heidelberg, Heidelberg, Germany; 17 Department of General, Visceral, Cancer and Transplantation Surgery, University Hospital of Cologne, Cologne, Germany; 18 Department of Gastroenterology and Hepatology, University Hospital of Heidelberg, Heidelberg, Germany; 19 Institute of Pathology, Heidelberg University Hospital, Heidelberg, Germany; 20 Department of Medical Oncology, National Center for Tumor Diseases (NCT), Heidelberg University Hospital, Heidelberg, Germany; 21 Clinical Cooperation Unit Applied Tumor-Immunity, German Cancer Research Center (DKFZ), Heidelberg, Germany; 22 Department of Medicine I, University Hospital Carl Gustav Carus, Dresden, Germany; 23 Center for Regenerative Therapies (CRTD), Technische Universität Dresden, Dresden, Germany; 24 Department of General, Visceral, and Thoracic Surgery, University Medical Center Hamburg-Eppendorf, Hamburg, Germany; 25 Tumour- and Normal Tissue Bank of the University Cancer Center (UCC), University Hospital Carl Gustav Carus, Medical Faculty, Technische Universität Dresden, Dresden, Germany

**Keywords:** Gastric cancer, Personalized medicine, Patient-derived organoids, Response prediction

## Abstract

**Background and aims:**

This study sought to determine the value of patient-derived organoids (PDOs) from esophago-gastric adenocarcinoma (EGC) for response prediction to neoadjuvant chemotherapy (neoCTx).

**Methods:**

Endoscopic biopsies of patients with locally advanced EGC (*n* = 120) were taken into culture and PDOs expanded. PDOs' response towards the single substances of the FLOT regimen and the combination treatment were correlated to patients' pathological response using tumor regression grading. A classifier based on FLOT response of PDOs was established in an exploratory cohort (*n* = 13) and subsequently confirmed in an independent validation cohort (*n* = 13).

**Results:**

EGC PDOs reflected patients' diverse responses to single chemotherapeutics and the combination regimen FLOT. In the exploratory cohort, PDOs response to single 5-FU and FLOT combination treatment correlated with the patients' pathological response (5-FU: Kendall's *τ* = 0.411, *P* = 0.001; FLOT: Kendall's *τ* = 0.694, *P* = 2.541e-08). For FLOT testing, a high diagnostic precision in receiver operating characteristic (ROC) analysis was reached with an AUC_ROC_ of 0.994 (CI 0.980 to 1.000). The discriminative ability of PDO-based FLOT testing allowed the definition of a threshold, which classified in an independent validation cohort FLOT responders from non-responders with high sensitivity (90%), specificity (100%) and accuracy (92%).

**Conclusion:**

In vitro drug testing of EGC PDOs has a high predictive accuracy in classifying patients' histological response to neoadjuvant FLOT treatment. Taking into account the high rate of successful PDO expansion from biopsies, the definition of a threshold that allows treatment stratification paves the way for an interventional trial exploring PDO-guided treatment of EGC patients.

**Supplementary Information:**

The online version contains supplementary material available at 10.1186/s12943-023-01919-3.

## Introduction

Gastric and esophageal cancers are among the most common and deadliest malignancies worldwide, ranking fifth and seventh in cancer incidence as well as fourth and sixth in cancer related death, respectively [1]. Perioperative chemotherapy has substantially improved the median survival in locally advanced gastric adenocarcinoma and adenocarcinoma of the esophago-gastric junction (combined in the following as esophago-gastric adenocarcinoma; EGC) patients to 50 months and a projected 5-year survival of 45% with the FLOT regimen (5-fluorouracil (5-FU), leucovorin, oxaliplatin and docetaxel) [2]. However, 63% of patients have no major pathological response (defined as < 10% vital tumor cells remaining in the resected primary tumor) or do not proceed to surgery after neoadjuvant chemotherapy (neoCTx) [3]. Pathological response to neoCTx has been shown to be a prognostic factor and correlate with survival in gastric cancer [4]. Individualization of neoCTx regimens to improve pathological response has the potential to improve patients' outcome.

Organoids are 3D cell culture models that allow in vitro tissue growth recapitulating many aspects of the original tissue [5]. This method has subsequently been optimized for human cancer tissue resulting in patient-derived organoid (PDO) biobanks from various tissues including EGC [6, 7]. PDOs demonstrated high phenotypic and molecular similarity to their respective tumor of origin, and in vitro treatment resulted in differential responses. These properties place PDOs in the spotlight of personalized treatment approaches [8]. A correlation between PDO and patient response could already be established for EGCs in selected cases [9]. Furthermore, for locally advanced rectal, pancreatic and metastatic colorectal cancer a predictive value of PDOs for therapy response could be demonstrated in prospective co-clinical trials [10].

In order to successfully individualize neoCTx for locally advanced EGC, it is critical to establish biomarkers that predict therapy response. Within the "Outcome prediction of systemic treatment in esophago-gastric carcinoma" (Opposite) study (ClinicalTrials.gov #NCT03429816) aiming at the identification of biomarkers correlating with treatment response, we prospectively established treatment naïve EGC-derived PDOs and investigated their ability to predict the pathological response to neoadjuvant FLOT therapy.

## Results

**Fig. 1:**
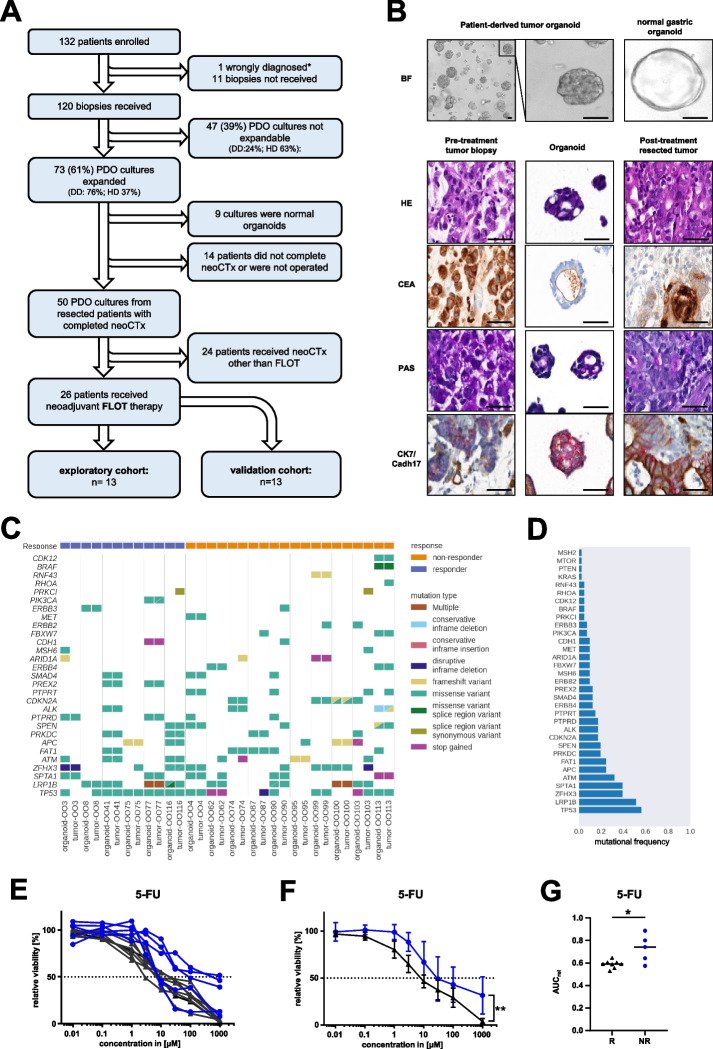
Trial flow chart and phenotypic, molecular as well as functional characterization of PDO cohort. **A** Flow chart of the study population. DD/HD: University hospital of Dresden (DD) and Heidelberg (HD). **B** Brightfield images of a representative EGC organoids line (OO4) and a normal gastric PDO line as a comparison (scale bar: 50 µm). Representative images of the histopathological characterization of primary tumor and corresponding PDO (OO4). The treatment naïve tumor biopsy, thereof derived organoid culture and the post-treatment resection specimen were characterized by hematoxylin and eosin (HE) staining, carcinoembryonic antigen (CEA) staining, periodic acid-Schiff reaction (PAS) as well as cytokeratin7 and cadherin17 (CK7/CADH17) double-staining (scale bar: 50 μm). **C** Oncoplot depicting prevalent genetic alterations in primary tumors and derived PDO lines. **D** Bar graph of mutational frequencies of this study. **E** Dose response curves from cell viability assay of 5-FU treated PDOs 144 h post treatment (average of *n* = 3 replicates per PDO). **F** Combined dose response curves from PDOs according to the patients' pathological response of 5-FU with standard deviations (repeated measures analysis of variance (ANOVA) of grouped PDOs, *P* = 0.001 for 5-FU). *P < 0.05; **P < 0.01. **G** Comparison of the relative area under the curve (AUC_rel_) of PDOs grouped by the patients' pathological response (R: responder; NR: non-responder) for 5-FU (unpaired two-tailed student's t-test, *P* = 0.010 for 5-FU, *P < 0.05). Blue lines represent pathological non-responders, black lines are pathological responders

EGC PDOs were generated according to a previously established protocol [7]. Overall, biopsies of 120 patients obtained from two different study sites Dresden (DD), and Heidelberg (HD) were taken into culture and 73 (61%) could be successfully expanded (Fig. 1A). From the successfully expanded PDOs, nine were found to be normal gastric organoids based on morphology, histopathological stainings, or long-term passaging capability. For further 14 PDOs, the corresponding patients did not complete neoCTx or undergo surgery. The pathological response to neoCTx was evaluated according to Becker et al*.* for the remaining patients (*n* = 50) [4]. PDOs from 26 patients received neoCTx according to the FLOT regimen and were further analyzed, while the remaining 24 patients split into small cohorts receiving a heterogeneous spectrum of other neoCTx regimens. The chronologically first 13 PDOs constituted the exploratory cohort, whereas the following 13 PDOs were used for independent validation. The analyzed cohort of 26 patients contained tumors from the esophago-gastric junction and the stomach, representing the whole spectrum of patients undergoing neoadjuvant treatment according to current clinical guidelines (Suppl. Table 1 and 2).

The overall successful culture rate for all 120 patients was 61% (Suppl. Figure 1A), with a rate of 76% at the study site with experience in EGC PDO generation (DD) and an improvement for the second study site (HD) over time after an initial implementation phase and subsequently continued optimization of workflows (Suppl. Figure 1A). Of note, the histological subtype according to Lauren influenced the success rate of the PDO generation (Suppl. Table 3). PDOs grew better from the mixed or diffuse subtype than from the intestinal subtype. The first major objective of the study was to secure established PDO lines by cryopreservation. Thus, the median time from receipt of the biopsy to the first FLOT test result can only be assessed retrospectively. The first results from two independent FLOT tests could have been available 20 days after direct processing and 62 days after delivery from the second study site after biopsy collection (Suppl. Figure 1B).

Established PDOs exhibited distinct morphologies differentiating them from the normal single-layered gastric organoids: several PDOs showed a cystic structure with a large lumen, some are characterized by a compact structure and no or small lumina, while others present with a diffuse morphology with poorly cohesive cell growth (Fig. 1B, Suppl. Figure 2). Immunohistochemistry confirmed the preservation of typical gastric cancer markers in PDOs such as cytokeratin 7 (CK7), cadherin 17 (CDH17), carcinoembryonic antigen (CEA) and periodic acid Schiff reaction (PAS) (Fig. 1B). Molecular characterization revealed a high mutational concordance between PDO and primary tumor (Fig. 1C). Additionally, the presence of typical mutations and copy number alterations of EGC in comparable frequency to published data sets indicates the representativeness of the analyzed cohort (Fig. 1D, Suppl. Figure 3, Suppl. Figure 4) [11].

First, PDOs of the exploratory cohort were analyzed with regard to their response to the single FLOT components 5-FU, oxaliplatin and docetaxel using cell viability assays (Fig. 1E-G, Suppl. Figure 5A-F). PDOs showed differential responses to the three chemotherapeutics in dose–response curves (DRCs) (Fig. 1E, Suppl. Figure 5A + D). While some PDOs showed a reduced viability already at low drug concentrations, others only responded at higher concentrations. Furthermore, some PDOs maintained a certain viability plateau at higher doses, e.g. three lines treated with 5-FU still displayed approximately 50% viability at the highest concentration, potentially indicating the presence of a resistant subpopulation. When PDOs were grouped by the patients' pathological regression grade of the resection specimen into responders (Becker 1a/b; *n* = 8) or non-responders (Becker 2/3; *n* = 5) [4], no difference in the grouped DRCs could be detected for oxaliplatin and docetaxel (Suppl. Figure 5B + E). In contrast, a significant difference between the two groups was revealed for 5-FU (repeated measures analysis of variance (ANOVA), *P* = 0.001) (Fig. 1F). In an independent analysis, relative area under the curve (AUC_rel_) values of the DRCs were calculated and PDOs of pathological responders and non-responders compared using a parametric analysis of non-maximal responses. While AUC_rel_ values of oxaliplatin or docetaxel did not differ between the two groups (Suppl. Figure 5C + F), AUC_rel_ values of 5-FU were again significantly distinct (unpaired two-tailed student's *t*-test, *P* = 0.010) (Fig. 1G).

**Fig. 2:**
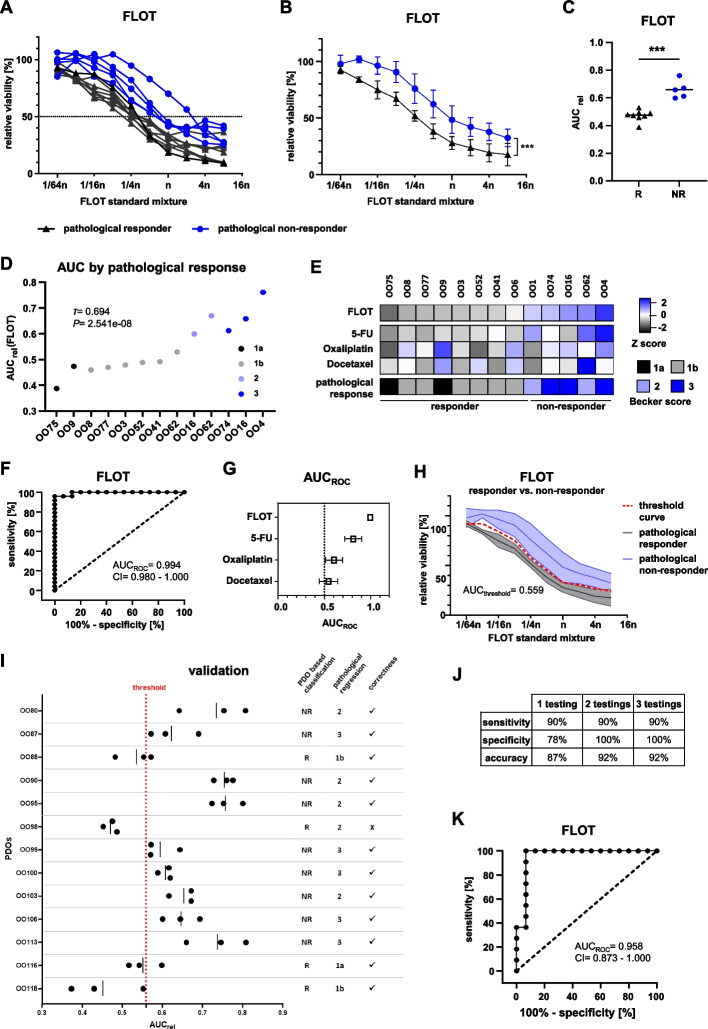
Establishment and validation of an in vitro threshold value to predict in vivo FLOT response. **A** Dose response curves (DRC) from cell viability assay of PDOs treated with the standard FLOT mixture (n) in varying dilutions analyzed 144 h post treatment (average of *n* = 3 replicates per PDO). **B** Combined FLOT-DRC from PDOs according to the patients' pathological response after FLOT neoCTx with standard deviations (repeated measures analysis of variance (ANOVA) of grouped PDOs, *P* = 4.55e-06). ***P < 0.001 **C**, Comparison of the relative area under the curve (AUC_rel_) of PDOs grouped by the patients' pathological response (R: responder; NR: non-responder) (two-tailed student's *t*-test, *P* = 3.94e-05). ***P < 0.001 **D** Dot plot of AUC_rel_(FLOT) ordered by the patients’ pathological response. Correlation analysis was performed using Kendall ordinal correlation. *P* < 0.005 was considered statistically significant. **E** Heatmap of the patients' pathological response and AUC_rel_ derived Z scores from single drug and FLOT combination treatment. Color scale indicates Z score (± 2.5; -2.5: black, 0: white, + 2.5: blue) and scored patients' pathological response according to Becker et al. **F** ROC curve of the AUC_rel_(FLOT) generated from single FLOT treatments of PDOs (*n* = 39, 3 replicates of 13 PDOs). **G** Summary graph of the AUC_rel_(ROC) and confidence interval (CI) 95% for FLOT, 5-FU, oxaliplatin and docetaxel. **H** Dose response curve with CI of FLOT combination treatment grouped according to the patients' pathological response with calculated threshold curve and calculated AUC_threshold_(FLOT). Blue lines represent pathological non-responders, black lines are pathological responders. **I** FLOT combination test results with subsequent classification according to the determined threshold value (AUC_threshold_ = 0.559) into responding (R) and non-responding (NR) patients. Correctness of classification was later assessed by comparison to patients' pathological response. Red dotted line indicates the threshold established in the exploratory cohort. **J** Achieved sensitivity, specificity and accuracy depending on the number of performed FLOT testings considered for classification. **K** ROC curve of the AUC_rel_(FLOT) generated from the mean of three independent replicates for each PDO of the whole cohort (*n* = 26)

Based on the single drug assays, a combination drug assay for all three drugs plus calcium folinate (FLOT) was established (see Supplementary Material, Material and Methods). PDO treatment with FLOT resulted in diverging DRCs, indicating a varying sensitivity (Fig. 2A). Notably, the PDOs with the lowest sensitivity to FLOT were derived from pathologically non-responding patients.

As already seen in the single drug treatments, the viability of several PDOs continuously dropped only to a certain level of maximum response, with no further decrease at higher FLOT concentrations. Of note, most PDOs that showed this phenomenon were derived from patients with a substantial amount of residual tumor in the resection specimen (Becker 2/3). The comparison of grouped curves showed a significantly higher mean viability of the PDOs from non-responding compared to responding patients (repeated measures ANOVA, *P* = 4.55e-06) (Fig. 2B). In line with this, the AUC_rel_ of PDOs from responders was significantly lower than the AUC_rel_ of PDOs from non-responders (unpaired student's *t*-test, *P* = 3.94e-05, average difference 0.18 AUC_rel_) (Fig. 2C). Accordingly, there was a strong positive association in ordinal correlation analysis between treatment response of PDOs and patients' pathological regression grade (Kendall's *τ* = 0.694, *P* = 2.541e-08) (Fig. 2D). A less strong association was found for single 5-FU (Kendall's *τ* = 0.411, *P* = 0.001), while no significant correlation was observed for oxaliplatin and docetaxel (Suppl. Figure 6A-C). A heatmap of Z scores revealed similar patterns between patients' pathological response and the PDOs response to FLOT (Fig. 2E).

Receiver operating characteristic (ROC) curves were generated to determine the diagnostic ability of PDOs to classify patients into pathological responders and non-responders (Fig. 2F, Suppl. Figure 6D-F). For FLOT an AUC_ROC_ of 0.994 (CI 0.980 to 1.000) was calculated from individual treatments (3 replicates of 13 PDOs = 39 measurements), demonstrating the high discriminatory ability of the PDO cultures to predict the patients' response to neoadjuvant FLOT treatment. The AUC_ROC_ of FLOT thereby outperformed the AUC_ROC_ of single substances (Fig. 2G). In order to determine a threshold for in vitro discrimination of responders vs. non-responders, a threshold curve was calculated in-between the confidence interval (CI) of grouped curves of responders and non-responders (Fig. 2H). From this threshold curve the AUC_threshold_ was calculated to be 0.559. Applying this threshold, all patients from the exploratory cohort were correctly classified (Fig. 2C).

The validity of the established threshold was then evaluated using an independent validation cohort (Fig. 2I). The threshold correctly classified 12 out of 13 patients and achieved a sensitivity of 90%, a specificity of 100% and an accuracy of 92% when PDOs were tested at least twice (Fig. 2J). Three replicates per PDO did not further improve the classification. In addition, the overall good discriminatory ability of the PDO test system could be demonstrated by a ROC analysis of the whole cohort resulting in a high AUC_ROC_ of 0.958 (CI: 0.873–1.000) (Fig. 2K). Notably, no other patient or tumor characteristic showed any discriminating value (Suppl. Table 4). Furthermore, no specific mutation could be correlated with the drug response to FLOT (Suppl. Table 5).

An issue frequently observed by several labs during EGC PDO generation that negatively impacts their use is the contamination by normal gastric organoids [12]. In the validation cohort, only one patient (OO98) was incorrectly classified. Detailed histological analysis over time revealed heterogeneity within the PDO culture (Suppl. Figure 7A). In low passages, the dominant PDO phenotype was cystic with a large lumen, resembling normal gastric organoids. Opposed to this, the phenotype in higher passages switched to a diffuse growing morphology, which is in line with primary tumor characteristics (Suppl. Table 2). When subjected to FLOT testing, the higher passage of OO98 was classified as a non-responder, which is the correct classification according to the pathological regression grade (Suppl. Figure 7B). This passage-dependent behavior, only observed in case of OO98, was most likely the result of a contamination of the initially tested early passage with normal gastric organoids.

## Discussion

In this prospective co-clinical trial, a strong association of the in vitro PDO response to FLOT and the patient's pathological regression grade after neoadjuvant FLOT could be documented. The limitation of the presented study is the relatively small sample size. Nevertheless, PDO response curves allowed the establishment of an AUC-based threshold, which was subsequently validated in an independent cohort to discriminate with high sensitivity, specificity and accuracy responders from non-responders. Interestingly, 5-FU, as a single drug, associated already well with the patients' pathological response, which is in line with clinical reports [13]. However, the inclusion of all FLOT components is required to achieve the highest predictive value. In case of FLOT non-response, in vivo studies, for example in orthotopic human PDO xenograft models, could serve as a validation platform for novel therapeutic strategies derived from PDO drug screening, which cannot be correlated to the patients’ in vivo response.

Prerequisites for implementing PDO drug testing into clinical decision-making are firstly a high rate of successful culture establishment and secondly a quick expansion of biopsy material to allow drug testing within a few weeks. Within this trial, the overall successful culture rate was 76% at the study site with experience in EGC PDO generation. This demonstrates that generation of PDOs from endoscopic biopsies followed by drug testing is feasible in more than two-thirds of patients in the clinical setting. In addition, the outgrowth rate for biopsies that were shipped increased over time and reached 50% at the end of the recruitment phase. With further improvements of delivery times (> 24 h is detrimental for culture establishment in our hands), multi-centric studies using EGC PDOs seem possible.

For clinical application, the required time to obtain in vitro test results has been a discussion point in PDO-guided therapy [10, 14]. In the current study, the primary focus during the initial expansion of the PDOs was to generate enough material for cryopreservation of the organoid line. Since the material necessary for one FLOT testing corresponds to one-third of what is needed for cryopreservation, it can be assumed that the test results would have been be available within three weeks for biopsies processed on the same campus. The treatment start for most patients in this study was two to three weeks after endoscopy. We expect that the time-to-result could decrease significantly by optimizing the protocol, i.e., miniaturizing the plate format, focusing on immediate testing of the PDOs and optimized sample transport times for external biopsies [15]. Therefore, it can be assumed that the result of FLOT testing could be available at a time that does not delay the start of neoCTx in a clinically relevant way. In addition, systemic therapy could be started with a standard chemotherapy regime and adjusted once the results of the PDO testing are available.

## Conclusion

The current study is the first to define and validate a PDO-derived threshold value to classify the patients' pathological response with high accuracy. The defined threshold for FLOT, in combination with future PDO-based alternative drug screens in the case of FLOT non-response allow now the design of interventional organoid-guided clinical trials for EGC patients. These trials will need to clarify the impact of organoids on oncological outcome parameters as well as on patient-reported outcome measures.

### Supplementary Information

**Additional file 1.** 

## Data Availability

Data and analytical methods are available to other researchers upon request to the corresponding author. Patient derived organoid models can be made available upon approval of the local ethics committee.

## Abbreviations

ANOVA: Analysis of variance
AUC: Area under the curve
AUC_rel_: Relative area under the curve
CI: Confidence interval
DRC: Dose–response curve
EGC: Esophago-gastric adenocarcinoma
FLOT: 5-Fluorouracil, leucovorin, oxaliplatin and docetaxel
neoCTX: Neoadjuvant chemotherapy
PDO: Patient-derived organoid
ROC: Receiver operator characteristics
5-FU: 5-Fluorouracil

## References

[1] Sung H, Ferlay J, Siegel RL, Laversanne M, Soerjomataram I, Jemal A (2021). Global Cancer Statistics 2020: GLOBOCAN Estimates of Incidence and Mortality Worldwide for 36 Cancers in 185 Countries. CA Cancer J Clin.

[2] Al-Batran S-E, Homann N, Pauligk C, Goetze TO, Meiler J, Kasper S (2019). Perioperative chemotherapy with fluorouracil plus leucovorin, oxaliplatin, and docetaxel versus fluorouracil or capecitabine plus cisplatin and epirubicin for locally advanced, resectable gastric or gastro-oesophageal junction adenocarcinoma (FLOT4): a ra. Lancet.

[3] Al-Batran SE, Hofheinz RD, Pauligk C, Kopp HG, Haag GM, Luley KB (2016). Histopathological regression after neoadjuvant docetaxel, oxaliplatin, fluorouracil, and leucovorin versus epirubicin, cisplatin, and fluorouracil or capecitabine in patients with resectable gastric or gastro-oesophageal junction adenocarcinoma (FLOT4-AIO. Lancet Oncol.

[4] Becker K, Mueller JD, Schulmacher C, Ott K, Fink U, Busch R (2003). Histomorphology and grading of regression in gastric carcinoma treated with neoadjuvant chemotherapy. Cancer.

[5] Sato T, Stange DE, Ferrante M, Vries RGJ, Van Es JH, Van Den Brink S, et al. Long-term expansion of epithelial organoids from human colon, adenoma, adenocarcinoma, and Barrett’s epithelium. Gastroenterology. 2011;141(5):1762–72. Available from: 10.1053/j.gastro.2011.07.050 10.1053/j.gastro.2011.07.050 21889923

[6] Bartfeld S, Bayram T, Van De Wetering M, Huch M, Begthel H, Kujala P, et al. In vitro expansion of human gastric epithelial stem cells and their responses to bacterial infection. Gastroenterology. 2015;148(1):126–136.e6. Available from: 10.1053/j.gastro.2014.09.042 10.1053/j.gastro.2014.09.042 PMC4274199 25307862

[7] Seidlitz T, Merker SR, Rothe A, Zakrzewski F, Von Neubeck C, Grützmann K (2019). Human gastric cancer modelling using organoids. Gut.

[8] Van De Wetering M, Francies HE, Francis JM, Bounova G, Iorio F, Pronk A, et al. Prospective derivation of a living organoid biobank of colorectal cancer patients. Cell. 2015;161(4):933–45. Available from: 10.1016/j.cell.2015.03.053 10.1016/j.cell.2015.03.053 PMC6428276 25957691

[9] Vlachogiannis G, Hedayat S, Vatsiou A, Jamin Y, Fernández-Mateos J, Khan K, et al. Patient-derived organoids model treatment response of metastatic gastrointestinal cancers. Science. 2018;359(6378):920 LP – 926. Available from: http://science.sciencemag.org/content/359/6378/920.abstract 10.1126/science.aao2774 PMC6112415 29472484

[10] Wensink GE, Elias SG, Mullenders J, Koopman M, Boj SF, Kranenburg OW, et al. Patient-derived organoids as a predictive biomarker for treatment response in cancer patients. NPJ Precis Oncol. 2021;5(1). Available from: 10.1038/s41698-021-00168-1 10.1038/s41698-021-00168-1 PMC8042051 33846504

[11] Bass AJ, Thorsson V, Shmulevich I, Reynolds SM, Miller M, Bernard B, et al. Comprehensive molecular characterization of gastric adenocarcinoma. Nature. 2014;513(7517):202–9. Available from: 10.1038/nature13480 10.1038/nature13480 PMC4170219 25079317

[12] Nanki K, Toshimitsu K, Takano A, Fujii M, Shimokawa M, Ohta Y, et al. Divergent routes toward Wnt and R-spondin Niche independency during human gastric carcinogenesis. Cell. 2018;174(4):856–869.e17. Available from: 10.1016/j.cell.2018.07.027 10.1016/j.cell.2018.07.027 30096312

[13] Culy CR, Clemett D, Wiseman LR (2000). Oxaliplatin: A review of its pharmacological properties and clinical efficacy in metastatic colorectal cancer and its potential in other malignancies. Drugs.

[14] Bose S, Clevers H, Shen X. Promises and challenges of organoid-guided precision medicine. Med. 2021;2(9):1011–26. Available from: 10.1016/j.medj.2021.08.005 10.1016/j.medj.2021.08.005 PMC8492003 34617071

[15] Gao M, Harper MM, Lin M, Qasem SA, Patel RA, Mardini SH, et al. Development of a single-cell technique to increase yield and use of gastrointestinal cancer organoids for personalized medicine application. J Am Coll Surg. 2020. 10.1016/j.jamcollsurg.2020.11.009 PMC8005421 33253861

