# Supplementary material for: Stratifying esophago-gastric cancer treatment using a patient-derived organoid-based threshold
Source: Mol Cancer. 2024 Jan 10;23:10. doi: 10.1186/s12943-023-01919-3 (PMC10777586; doi:10.1186/s12943-023-01919-3)
Supplement: Supplementary file 1 — Additional file 1. [file 12943_2023_1919_MOESM1_ESM.docx]

**Stratifying esophago-gastric cancer treatment using a patient-derived organoid-based threshold**

**Supplementary Material**

**Materials and Methods**

**Samples**

Endoscopic biopsies (2-3 pieces) were obtained before neoCTx from the primary tumor. Tissue samples in Dresden were kept in ice-cold NaCl solution until processing to PDOs, while samples from Heidelberg were stored in MACS® Tissue Storage Solution (Miltenyi Biotec) and send at 4°C to the Dresden laboratory.

**PDO generation and cultivation**

PDOs were generated and cultivated according to a previously published protocol(1). Endoscopic biopsies were minced, enzymatically digested using dispase II (Roche) and collagenase XI (Sigma-Aldrich), washed three times with antibiotics-containing medium (DMEM/F12, 10 mM HEPES (Thermo Fisher Scientific), 1x GlutaMAX™ (Thermo Fischer Scientific), 100 μg/ml Primocin (Invivogen)) and seeded in Matrigel (Corning). Gastric organoid medium (50% WNT-conditioned medium, 10% R-spondin-conditioned medium, 10% Noggin-conditioned medium, 1x B27 (Invitrogen), 10 mM nicotinamide (Sigma-Aldrich), 1.25 mM N-acetyl-L-cysteine (Sigma-Aldrich), 200 ng/ml Fgf10 (Preprotech), 50 ng/ml Egf (Invitrogen), 0.5 µM A83-01 (Tocris Bioscience), 2 mM HEPES (Thermo Fisher Scientific), 0.25x GlutaMAX™ (Thermo Fisher Scientific), 100 μg/ml Primocin (Invivogen), 200 U/ml penicillin and 20 µg/ml streptomycin (Gibco Life Technologies) in DMEM/F12) was overlaid and supplemented with 10 µM Y-27632 (Sigma-Aldrich) for the first 2-6 passages. Depending on the growth rate, organoids were passaged up to twice a week with a ratio of 1:1.5 to 1:3.

The time course of PDO expansion was analyzed reterospectively in order to evaluate the feasibility of PDO FLOT testing within a clinically relevant time frame. To this end, we determined the number of days required to expand each PDO culture to the point where there would have been sufficient material to initiate FLOT testing, plus additional material to ensure further maintenance of the PDO culture. From the date of the start of the PDO culture to the potential start of the FLOT testing plus 7 days (duration of the FLOT test) estimates the time required to obtain the test results.

**Histopathological analysis**

PDOs were harvested from Matrigel using Cell Recovery Solution (Corning) and washed with Dulbecco's PBS. Recovered organoids were fixed for 10 min at room temperature using 4% formaldehyde solution, embedded into paraffin and cut into 3 µm sections.

Staining of organoid and primary tumor sections for HE, carcinoembryonic antigen (CEA), cytokeratin 7 (CK7), cadherin 17 (CADH17) and periodic acid Schiff (PAS) reaction was performed according to standard protocols. Stainings were scanned by a Digital Pathology Scanner (Philips Amsterdam, The Netherlands).

**PDO treatment and cell viability analysis**

To prepare cell viability assays, 1 well of a 48-well plate was split into 20 wells of a 384-well plate in 15 µl 50% Matrigel (Corning) per well. After solidification, PDOs were overlaid with gastric organoid medium. 24 h after seeding, the medium was replaced with medium containing the chemotherapeutics 5-FU, oxaliplatin and docetaxel at desired concentrations. The concentration range was optimized for each drug such that the DRCs center on the concentration with the widest variation. After 72 h of incubation, the medium containing chemotherapeutics was refreshed. Cell viability was measured after a total treatment time of 144 h. PDOs were incubated for 3 h at 37°C with Presto Blue Cell Viability Reagent (Invitrogen), and fluorescence measured at 560/590 nm using a Varioscan Lux (Thermo Fisher Scientific).

The mean log_10_IC50 values of three replicates were determined for each single chemotherapeutic using a cohort of PDOs (n= 13). A standard FLOT mixture (n) was set at mean IC50 values of 5‑FU (10.3 µM), oxaliplatin (10.7 µM) and docetaxel (1.2 nM). The FLOT mixture was used in a 1:2 dilution series from (8n to 1/64n) with a constant concentration of calcium folinate (10 µM) for FLOT drug assays. For combinatorial testing of FLOT components, one or two drugs were left out of the FLOT standard mixture.

Every PDO drug treatment was performed three times in independent experiments, and replicates were averaged for DRCs and subsequent analyses except for ROC curves, where individual experiments were used.

**Pathological regression grade**

The histological regression in the resected primary tumor was categorized according to Becker *et al.* (2003)(2). The pathological regression grades Becker 1a and 1b were classified as responders, while the grades Becker 2 and 3 were classified as non-responders. For subsequent statistical analyses, the regression grade was scored (Becker 1a: 1.0; 1b: 1.25; 2: 2.0; 3: 3.0).

**Mutational analysis**

In order to evaluate the representative character of the studied PDO cohort, PDO and primary tumor DNA was used for targeted sequencing using TruSight Oncology 500 Kit (Illumina). Therefore, 120 ng DNA per sample were subjected to library preparation according to the manufacturer's protocol. Data were processed as previously described(3).

Frequencies of gene mutations (non-synonymous) within analyzed PDO cohort were compared to the publicly accessible TCGA Firehose data set for stomach adenocarcinoma (n= 478)(4) samples and esophageal adenocarcinoma (n= 89)(5) accessed via cBioPortal for Cancer Genomics (http://cbioportal.org)(6,7). For comparison, only genes with a mutation frequency ≥ 10% were considered and plotted with ascending mutation frequency.

The copy number alterations were analysed for genes covered by the TSO500 panel and having a CNA frequency of ≥ 5% for stomach or ≥ 10% for esophageal adenocarcinoma (TCGA Firehose data set)(4,5).

**Statistical analysis**

Each PDO line was analyzed in three independent drug assays. Values of the independent experiments were averaged, the standard deviation and confidence interval (CI) calculated, DRCs generated and Log_10_IC50 and AUC_DRC_ values determined using Prism (GraphPad 8.4.0; GraphPad, La Jolla, CA). AUC_rel_ is the quotient of AUC_DRC_ normalized to AUC_100%_ (relative viability equal to 100%).

Grouped DRCs were compared using Prism two-way repeated measures analysis of variance (ANOVA) (**P*< 0.05; ***P*< 0.01; ****P*< 0.001).

AUC_rel_ values of the grouped PDOs were compared using unpaired two-tailed Student's *t*-test (**P*< 0.05; ***P*< 0.01; ****P*< 0.001).

AUC_rel_ of PDOs' single and combination drug assays were tested for correlation with the scored regression grade using Kendall's tau ordinal correlation coefficient with *P*< 0.005 considered statistically significant (multiple comparison adjustment).

To determine a threshold for *in vitro* FLOT testing discriminating responder vs. non-responding patients, a mean DRC with confidence intervals (CI) was generated for each group. Next, the mean viability at each concentration between the lower CI of non-responders and the higher CI of responders was calculated. The AUC of the resulting DRC was determined and represents the threshold between the responders and non-responders (AUC_threshold_).

ROC curves and resulting AUC_ROC_ with corresponding confidence intervals were generated and analyzed using Prism (GraphPad 8.4.0; GraphPad, La Jolla, CA). The threshold validation was performed in the R Environment for Statistical Computing.

Univariate analyses for the impact of patient or tumor characteristics on organoid expansion and response was performed using binary logistic regression. The significance level was set at p=0.05. SPSS (version 28.0.1.0, IBM Corp., Armonk, NY, USA) was used for statistical analysis

**Supplementary Figures**


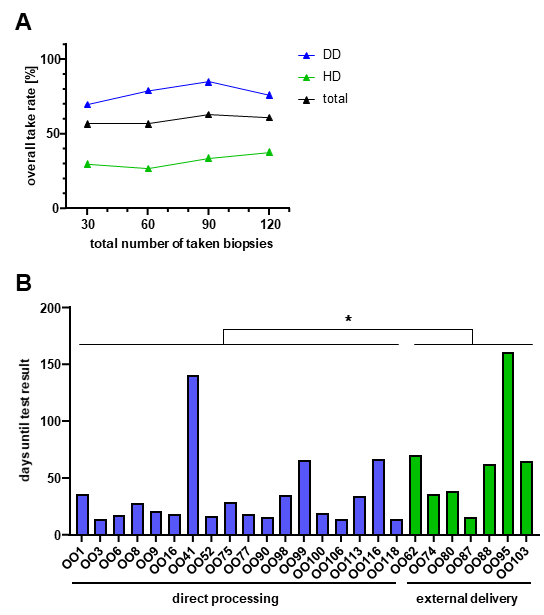


**Supplementary Figure 1:** Culture rate and time to test result of biopsy-derived EGC PDOs.

**A**, Percentage of successfully expanded PDOs (culture rate) for the study sites Dresden (DD) and Heidelberg (HD). The overall culture rate of PDOs was calculated for the first 30, 60, 90 and 120 PDOs. **B**, Time to first potential FLOT test result analysed retrospectively, for biopsy-derived PDOs at the Dresden site (direct processing, n= 18) and the Heidelberg site (external biopsy with shipment, n= 7) (unpaired two-tailed student's *t*-test, *P*= 0.045). **P*< 0.05)

**
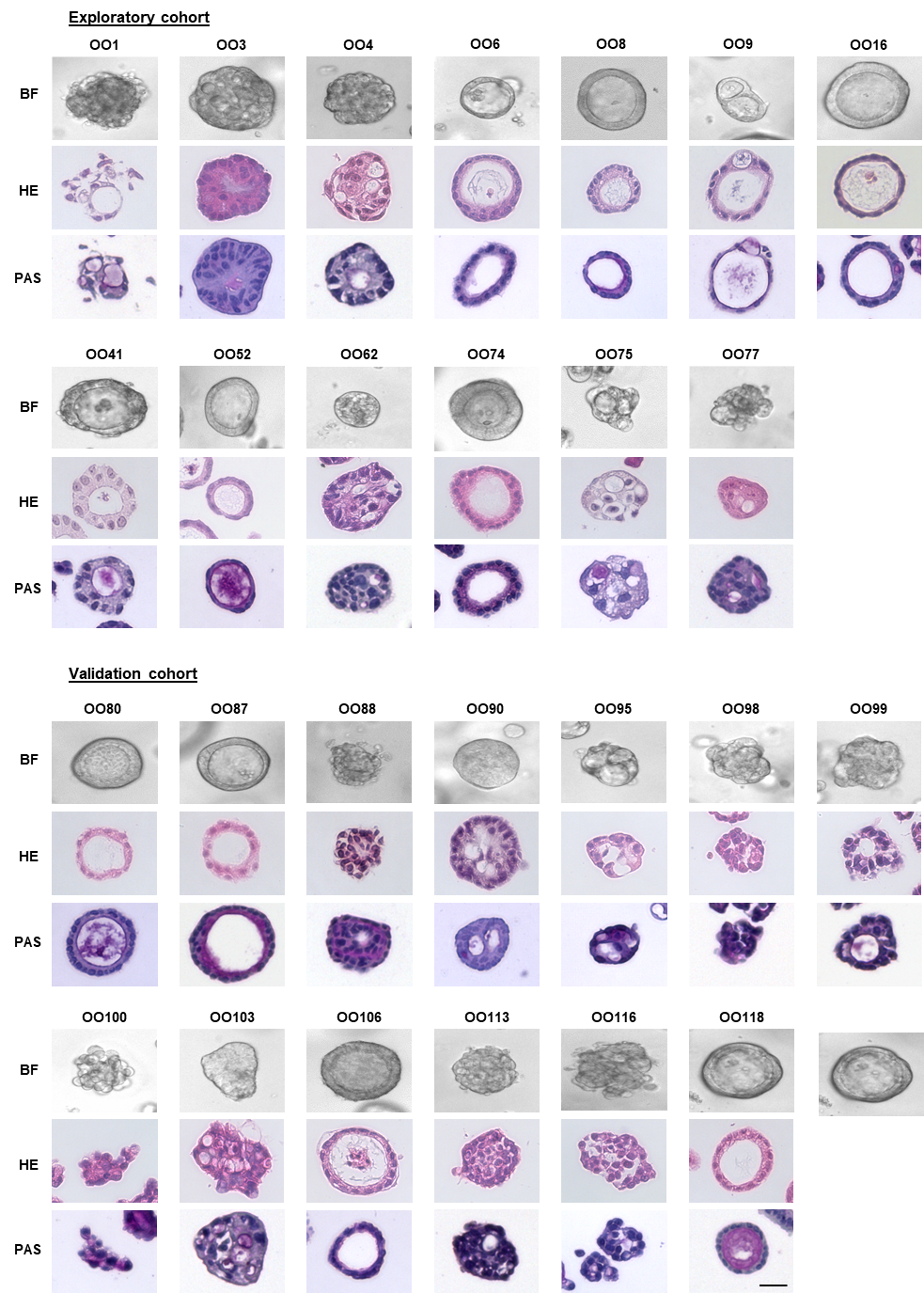
**

**Supplementary Figure 2:** Generated cohort of biopsy-derived EGC PDOs

Morphological characterization of the 26 EGC-PDO lines of the exploratory and validation cohort. Representative bright field images (BF), hematoxylin and eosin (HE) staining and periodic acid-Schiff reaction (PAS) staining (scale bar: 50 μm).


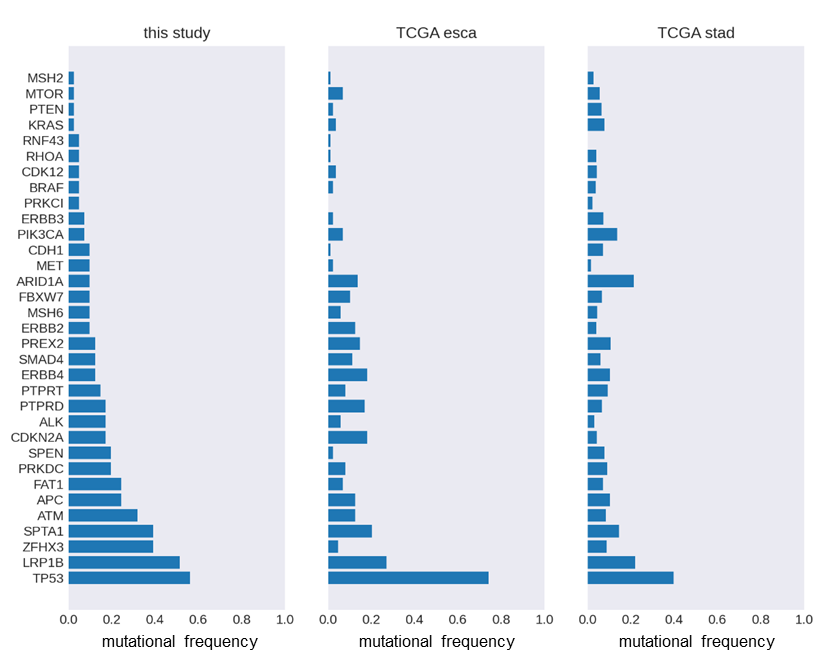


**Supplementary Figure 3:** Comparison of mutational frequencies to TCGA datasets.

Bar graph of mutational frequencies of this study and TCGA stomach adenocarcinoma (TCGA STAD) and esophageal adenocarcinoma (TCGA ESCA) data sets.(4–7)

**
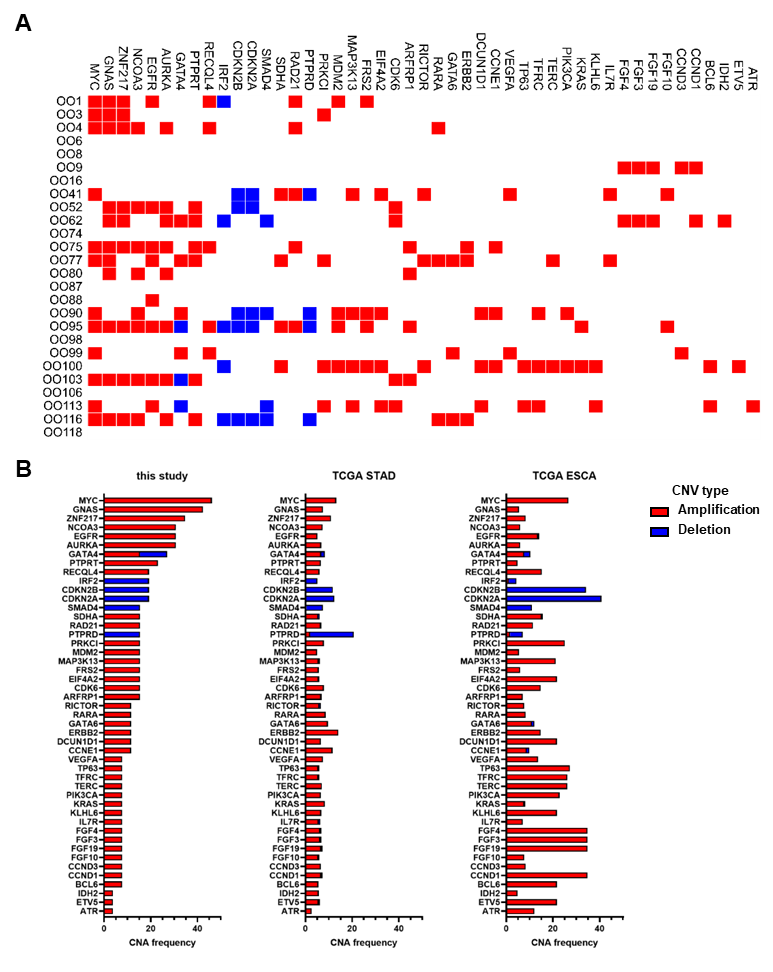
**

**Supplementary Figure 4:** Copy number alteration profile of PDOs and comparison to TCGA datasets.

**A**, Oncoplot depicting prevalent copy number alterations in the PDO lines. **B**, Bar graph of copy number alteration frequencies of this study and TCGA stomach adenocarcinoma (TCGA STAD) and esophageal adenocarcinoma (TCGA ESCA) data sets.


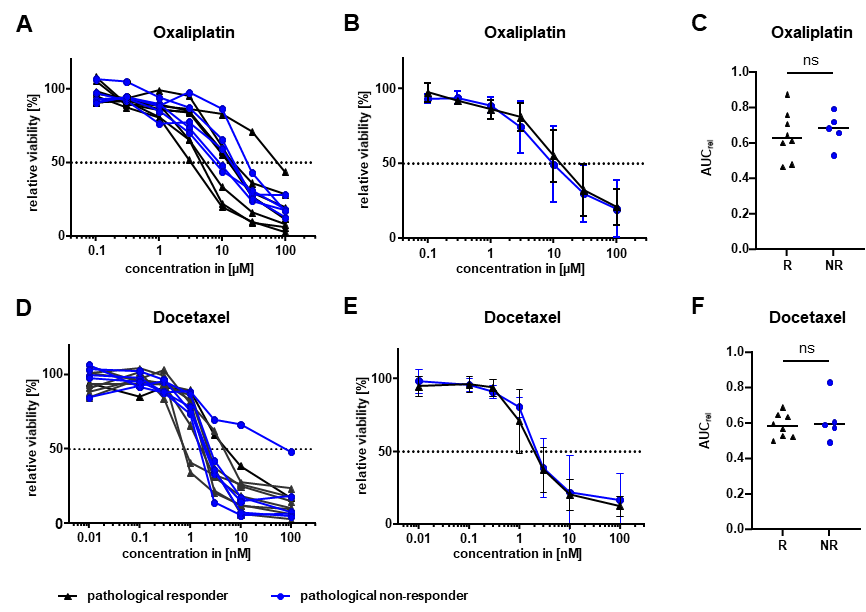


**Supplementary Figure 5:** Oxaliplatin and Docetaxel response testing.

**A**, **D**, Dose response curves from cell viability assay of oxaliplatin or docetaxel treated PDOs 144 h post treatment (average of n= 3 replicates per PDO). **B**, **E**, Combined dose response curves from PDOs according to the patients’ pathological response of oxaliplatin and docetaxel with standard deviations (repeated measures analysis of variance (ANOVA) of grouped PDOs). **C**, **F**, Comparison of the relative area under the curve (AUC_rel_) of PDOs grouped by the patients’ pathological response (R: responder; NR: non-responder) for oxaliplatin and docetaxel (unpaired two-tailed student’s t-test). Blue lines represent pathological non-responders, black lines are pathological responders.


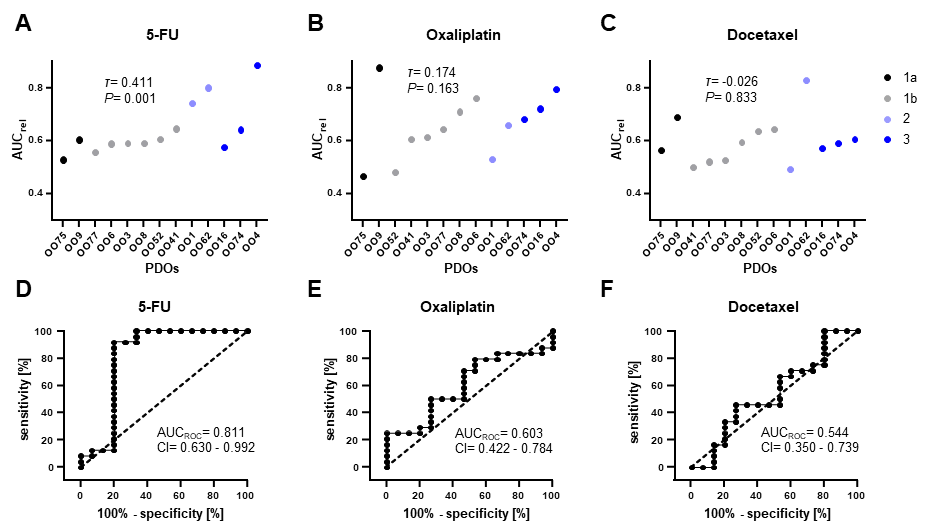


**Supplementary Figure 6:** FLOT single component drug testing.

**A-C**, Dot plot of AUC_rel_(5-FU), AUC_rel_(Oxaliplatin) and AUC_rel_(Docetaxel) ordered by the patients' pathological response. Correlation analysis was performed using Kendall ordinal correlation. *P*< 0.005 was considered statistically significant. **D-F**, ROC curve of the AUC_rel_ values generated from individual treatments of PDOs with 5-FU, oxaliplatin, docetaxel (n= 39, 3 replicates of 13 PDOs).


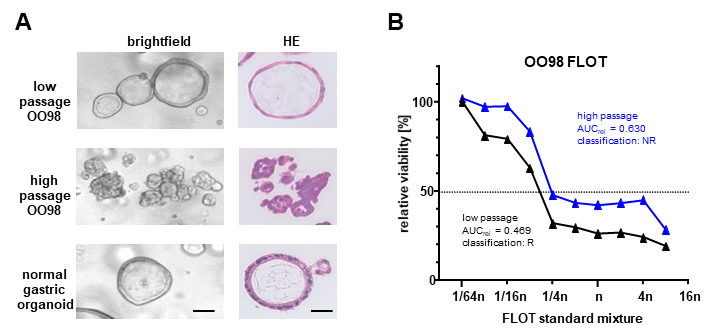


**Supplementary Figure 7:** Passage-dependent EGC PDO morphology and FLOT response classification (OO98).

**A**, Brightfield images and hematoxylin and eosin (HE) stainings of low and high passage OO98 PDOs and a normal gastric PDO line as a comparison. Scale bar: 50 µm. **B**, Dose response curves from cell viability assay of low and high passage OO98 treated with the FLOT standard mixture (average of n= 3 replicates). AUC_rel_ and resulting classifications into responder (R) or non-responder (NR) are depicted.

**Supplementary Tables**

| **Suppl. Table 1: Patient cohort characteristics** | | |  |
| --- | --- | --- | --- |
|  | **exploratory (n=13)** | **validation (n=13)** | **total (n=26)** |
|  |  |  |  |
| **Median age (years)** | 65 | 60 | 63 |
| **Sex** |  |  |  |
| male | 9 | 10 | 19 (73%) |
| female | 4 | 3 | 7 (27%) |
| **localization** |  |  |  |
| esophago-gastric junction | 9 | 8 | 17 (65%) |
| stomach | 4 | 5 | 9 (35%) |
| **Lauren classification*** |  |  |  |
| diffuse | 5 | 3 | 8 (33%) |
| intestinal | 6 | 6 | 12 (50%) |
| mixed | 2 | 2 | 4 (17%) |
| **TNM before FLOT** |  |  |  |
| **cT status** |  |  |  |
| T1/2 | 3 | 1 | 4 (15%) |
| T3/4 | 10 | 12 | 22 (85%) |
| **cN status** |  |  |  |
| N0 | 2 | 3 | 5 (19%) |
| N+ | 11 | 10 | 17 (81%) |
| **TNM after FLOT** |  |  |  |
| **ypT status** |  |  |  |
| T0 | 2 | 1 | 3 (11%) |
| T1/2 | 5 | 4 | 9 (35%) |
| T3/4 | 6 | 8 | 14 (54%) |
| **ypN status** |  |  |  |
| N0 | 9 | 6 | 15 (58%) |
| N+ | 4 | 7 | 11 (42%) |
| **pathological TRG**** |  |  |  |
| Ia | 2 | 1 | 3 (11%) |
| Ib | 6 | 2 | 8 (31%) |
| II | 2 | 5 | 7 (27%) |
| III | 3 | 5 | 8 (31%) |
| TNM: primary tumor (T), lymph node metastasis (N) and distant metastasis (M) staging; TRG: tumor regression grading  * for patient OO87 and OO88 not classifiable | | | |
| ** according to Becker *et al.* (2013) | |  |  |

| **Suppl. Table 2: Individual patient characteristics** | | | | | |  |  |  |
| --- | --- | --- | --- | --- | --- | --- | --- | --- |
|  | **Organoid** | **Age** | **Sex** | **Primary tumor localization** | **TNM before neoCTx** | **TNM after neoCTx** | **Grade of regression according to Becker et. al. (2003)** | **Lauren classification** |
|  |  |  |  |  |  |  |  |  |
| **exploratory cohort** | **OO1** | 79 | female | GAC | cT3 cN1 | ypT1b, ypN0 (0/16) | 2 | mixed |
|  | **OO3** | 65 | female | GAC | cT3 cN0 | ypT3, ypN2 (6/16) | 1b | diffuse |
|  | **OO4** | 59 | male | AEG | cT3 cN1 | ypT3 ypN0 (0/37) | 3 | mixed |
|  | **OO6** | 49 | female | GAC | cT1a cN+ | ypT3, ypN3b (24/29) | 1b | diffuse |
|  | **OO8** | 56 | male | AEG | cT3 cN2 | ypT2, ypN0 (0/31) | 1b | intestinal |
|  | **OO9** | 72 | male | AEG | cT4a cN1 | ypT0, ypN0 (0/29) | 1a | intestinal |
|  | **OO16** | 54 | male | GAC | cT3 cN+ | ypT4a, ypN2 (4/50) | 3 | intestinal |
|  | **OO41** | 65 | male | AEG | cT3 cN2 | ypT1b, ypN0 (0/13) | 1b | intestinal |
|  | **OO52** | 75 | female | AEG | cT3 cN+ | ypT3, ypN0 (0/17) | 1b | diffuse |
|  | **OO62** | 66 | male | AEG | cT2 cN1 | ypT1b, ypN0 (0/33) | 2 | intestinal |
|  | **OO74** | 57 | male | AEG | cT3 cN0 | ypT3, ypN2 (3/28) | 3 | diffuse |
|  | **OO75** | 67 | male | AEG | cT3 cN1 | ypT0, ypN0 (0/25) | 1a | diffuse |
|  | **OO77** | 68 | male | AEG | cT2 cN2 | ypT2, ypN0 (0/25) | 1b | intestinal |
| **validation cohort** | **OO80** | 52 | male | GAC | cT2 cN+ | ypT3, ypN0 (0/30) | 2 | mixed |
|  | **OO87** | 70 | male | AEG | cT3 cN+ | ypT3, ypN3b (16/34) | 3 | not classifiable |
|  | **OO88** | 60 | male | AEG | cT3 cN1 | ypT1, ypN0 (0/36) | 1b | not classifiable |
|  | **OO90** | 58 | male | GAC | cT3 cN+ | ypT1b ypN0 (0/23) | 2 | diffuse |
|  | **OO95** | 58 | male | AEG | cT3 cN1 | ypT3, ypN2 (6/34) | 2 | mixed |
|  | **OO98** | 41 | female | GAC | cT3 cN1 | ypT2, ypN1 (1/27) | 2 | diffuse |
|  | **OO99** | 78 | male | AEG | cT3 cN1 | ypT3, ypN1 (2/20) | 3 | intestinal |
|  | **OO100** | 50 | male | AEG | cT3 cN2 | ypT3, ypN3 (12/44) | 3 | intestinal |
|  | **OO103** | 67 | male | AEG | cT4a cN0 | ypT3, ypN1 (2/29) | 2 | intestinal |
|  | **OO106** | 70 | female | GAC | cT3 cN0 | ypT4a, ypN0 (0/22) | 3 | diffuse |
|  | **OO113** | 53 | male | AEG | cT3 cN1 | ypT4a, ypN2 (5/18) | 3 | intestinal |
|  | **OO116** | 62 | male | AEG | cT3 cN+ | ypT0 ypN0 (0/8) | 1a | intestinal |
|  | **OO118** | 63 | female | GAC | cT3 cN0 | ypT1b ypN0 (0/22) | 1b | intestinal |
| GAC: gastric adenocarcinoma; AEG: adenocarcinoma of the esophago-gastric junction; | | | | | | | | |
| TNM: primary tumor (T), lymph node metastasis (N) and distant metastasis (M) staging | | | | | | | | |

| **Suppl. Table 3: Univariate analysis for growth of PDOs** | | | |
| --- | --- | --- | --- |
| **n=120** | **univariate** | | |
|  | **OR** | **95% CI** | **p-value** |
| **age** | 1.025 | 0.984-1.067 | 0.231 |
| **sex (f : m)** | 0.78 | 0.315-1.932 | 0.592 |
| **BMI** | 1.004 | 0.882-1.143 | 0.951 |
| **smoking** | 0.427 | 0.123-1.476 | 0.179 |
| **alcohol** | 1.196 | 0.229-6.254 | 0.832 |
| **diabetes mellitus** | 0.68 | 0.180-2.575 | 0.57 |
| **ASA** | 0.727 | 0.410-1.290 | 0.276 |
| **localization  (EGJA : stomach)** | 2.562 | 0.873-7.518 | 0.087 |
| **cT stage** | 1.086 | 0.591-1.994 | 0.791 |
| **cN stage** | 1.346 | 0.688-2.633 | 0.386 |
| **Grading** | 0.806 | 0.288-2.254 | 0.681 |
| **Lauren type** | 2.174 | 1.102-4.287 | 0.025 |
| **Her2 neu status** | 1.703 | 0.580-5.001 | 0.333 |
| **MSI status** | 0.909 | 0.054-15.184 | 0.947 |
| BMI, body mass index; ASA, American Society of Anesthesiologists; EGJA, adenocarcinoma of the esophagogastric junction; binary logistic regression; MSI, microsatellite instability, OR: odds ratio, 95% CI: 95% confidence interval | | | |

| **Suppl. Table 4: Univariate analysis for response of PDOs** | | | |
| --- | --- | --- | --- |
| **n=26** | **univariate** | | |
|  | **OR** | **95% CI** | **p-value** |
| **age** | 1.034 | 0.946-1.130 | 0.46 |
| **sex (f : m)** | 0.438 | 0.075-2.552 | 0.358 |
| **BMI** | 1.107 | 0.813-1.507 | 0.518 |
| **smoking** | 1.75 | 0.129-23.703 | 0.674 |
| **alcohol** | 0.429 | 0.234-0.785 | 0.092 |
| **diabetes mellitus** | 0.5 | 0.306-0.816 | 0.477 |
| **ASA** | 2.333 | 0.336-16.180 | 0.391 |
| **localization  (EGJA : stomach)** | 0.563 | 0.105-3.023 | 0.502 |
| **cT stage** | 0.531 | 0.155-1.813 | 0.312 |
| **cN stage** | 1.813 | 0.521-6.305 | 0.349 |
| **Grading** | 0.8 | 0.118-5.404 | 0.819 |
| **Lauren type** | 2.334 | 0.226-24.085 | 0.477 |
| **Her2 neu status** | 3.6 | 0.257-50.330 | 0.341 |
| BMI, body mass index; ASA, American Society of Anesthesiologists; EGJA, adenocarcinoma of the esophagogastric junction; binary logistic regression; OR: odds ratio, 95% CI: 95% confidence interval | | | |

| **Supplementary Table 5: Gene mutation impact on drug response** | | | | | |
| --- | --- | --- | --- | --- | --- |
| mutated vs. wildtype* | number of mutated PDOs | **FLOT** (adjusted p value) | **5-FU** (adjusted p value) | **oxaliplatin** (adjusted p value) | **docetaxel** (adjusted p value) |
| *MET* | 3 | 1.000 | 1.000 | 0.993 | 1.000 |
| *ERBB2* | 3 | 1.000 | 0.997 | 0.985 | 1.000 |
| *MSH6* | 3 | 0.971 | 1.000 | 0.971 | 1.000 |
| *ERBB4* | 3 | 0.999 | 0.885 | 0.979 | 0.763 |
| *SMAD4* | 3 | 0.945 | 1.000 | 0.989 | 1.000 |
| *PREX2* | 3 | 0.547 | 0.547 | 0.833 | 0.995 |
| *PTPRT* | 3 | 0.885 | 0.999 | 0.995 | 1.000 |
| *CDKN2A* | 4 | 1.000 | 1.000 | 1.000 | 1.000 |
| *ALK* | 4 | 1.000 | 1.000 | 0.999 | 1.000 |
| *PTPRD* | 5 | 1.000 | 1.000 | 0.964 | 0.995 |
| *SPEN* | 4 | 0.999 | 0.907 | 0.970 | 0.320 |
| *PRKDC* | 5 | 1.000 | 0.999 | 0.993 | 0.961 |
| *APC* | 6 | 0.999 | 1.000 | 1.000 | 1.000 |
| *FAT1* | 7 | 0.964 | 1.000 | 1.000 | 1.000 |
| *ATM* | 7 | 1.000 | 1.000 | 0.993 | 1.000 |
| *ZFHX3* | 8 | 1.000 | 1.000 | 1.000 | 0.996 |
| *SPTA1* | 10 | 1.000 | 1.000 | 0.783 | 0.995 |
| *LRP1B* | 13 | 0.998 | 1.000 | 1.000 | 1.000 |
| *TP53* | 12 | 1.000 | 1.000 | 1.000 | 0.995 |
| *unpaired Mann-Whitney test (Holm-Šídák method), mutated vs. wildtype, 26 PDOs | | | | | |

**Additional references:**

1. Seidlitz T, Merker SR, Rothe A, Zakrzewski F, Von Neubeck C, Grützmann K, et al. Human gastric cancer modelling using organoids. Gut. 2019;68(2):207–17.

2. Becker K, Mueller JD, Schulmacher C, Ott K, Fink U, Busch R, et al. Histomorphology and grading of regression in gastric carcinoma treated with neoadjuvant chemotherapy. Cancer. 2003;98(7):1521–30.

3. Hennig A, Baenke F, Klimova A, Drukewitz S, Jahnke B, Brückmann S, et al. Detecting drug resistance in pancreatic cancer organoids guides optimized chemotherapy treatment. J Pathol. 2022;(May):607–19.

4. Bass AJ, Thorsson V, Shmulevich I, Reynolds SM, Miller M, Bernard B, et al. Comprehensive molecular characterization of gastric adenocarcinoma. Nature [Internet]. 2014;513(7517):202–9. Available from: http://dx.doi.org/10.1038/nature13480

5. Kim J, Bowlby R, Mungall AJ, Robertson AG, Odze RD, Cherniack AD, et al. Integrated genomic characterization of oesophageal carcinoma. Nature. 2017;541(7636):169–74.

6. Cerami E, Gao J, Dogrusoz U, Gross BE, Sumer SO, Aksoy BA, et al. The cBio Cancer Genomics Portal: An open platform for exploring multidimensional cancer genomics data. Cancer Discov. 2012;2(5):401–4.

7. Gao J, Aksoy BA, Dogrusoz U, Dresdner G, Gross B, Sumer SO, et al. Integrative Analysis of Complex Cancer Genomics and Clinical Profiles Using the cBioPortal Complementary Data Sources and Analysis Options. Sci Signal [Internet]. 2014;6(269):1–20. Available from: http://www.ncbi.nlm.nih.gov/pubmed/23550210%0Ahttp://www.pubmedcentral.nih.gov/articlerender.fcgi?artid=PMC4160307
